# People Who Die by Suicide Without Receiving Mental Health Services: A Systematic Review

**DOI:** 10.3389/fpubh.2021.736948

**Published:** 2022-01-18

**Authors:** Samantha Tang, Natalie M. Reily, Andrew F. Arena, Philip J. Batterham, Alison L. Calear, Gregory L. Carter, Andrew J. Mackinnon, Helen Christensen

**Affiliations:** ^1^Black Dog Institute, University of New South Wales, Sydney, NSW, Australia; ^2^Centre for Mental Health Research, Research School of Population Health, Australian National University, Canberra, ACT, Australia; ^3^School of Medicine and Public Health, University of Newcastle, Newcastle, NSW, Australia

**Keywords:** suicide, systematic review, healthcare utilization, coronial data, mental health services

## Abstract

**Introduction:**

The majority of people who die by suicide have not had contact with mental health professionals in the year prior to death. To date, this majority group has largely been ignored, with most existing research focusing on predictors of suicide such as past suicide attempts. Identifying the characteristics of people who die by suicide without receiving services, often with a fatal first attempt, is crucial to reduce suicide rates through guiding improvements to service pathways and “just in time” interventions.

**Methods:**

In this systematic review, PsycInfo, PubMed, CINAHL, and Web of Science were searched for peer-reviewed articles published from 1980 to 1st March 2021. Included studies examined predictors of non-receipt of formal mental health services among people who died by suicide. Data were extracted from published reports and the quality of included studies was assessed using a modified version of the Joanna Briggs Institute Checklist for Analytical Cross Sectional Studies. This review was registered with PROSPERO, CRD 42021226543.

**Results:**

Sixty-seven studies met inclusion criteria, with sample sizes ranging from 39 to 193,152 individuals. Male sex, younger or older age, and rural location were consistently associated with non-receipt of mental health services. People not receiving mental health services were also less likely to have a psychiatric diagnosis, past suicidal behavior or contact with general health services, and more likely to use violent means of suicide. There was some evidence that minority ethnicity and psychosocial stressors were associated with service non-receipt.

**Conclusion:**

People who die by suicide without receiving mental health services are likely to have diverse profiles, indicating the need for multifaceted approaches to effectively support people at risk of suicide. Identifying the needs and preferences of individuals who are at risk of suicide is crucial in developing new support pathways and services, and improving the quality of existing services.

**Systematic Review Registration:**

http://www.crd.york.ac.uk/PROSPERO/display_record.asp?ID=CRD42021226543.

## Introduction

Suicide is the leading cause of death for Australians aged 15–49 (1). Notably, over two-thirds of individuals who die from suicide do not receive professional mental health support (including seeing a general practitioner or mental health professional for mental health or substance use reasons) in the year prior to death. (2). Research has focused on understanding suicide risk factors [for reviews, see (3–5)], but not on identifying factors specifically for those who did not receive mental health services prior to suicide. Although it is not possible to obtain first-hand reports from people who have died by suicide, coronial data can provide insights into the characteristics of this population and guide opportunities for suicide prevention efforts by policymakers, researchers, and service providers.

The current systematic review provides a comprehensive overview of predictors of non-receipt of formal mental health services among people who died by suicide, consolidating work in existing reviews on predictors of service utilization among those with common mental health issues (6, 7). To our knowledge, only two other systematic reviews have examined predictors of service use in a suicidal population. A systematic review by Han et al. (8), which examined predictors of help-seeking across different levels of suicidality, found that help-seeking for suicidality was associated with female gender, older age, non-minority ethnicity, presence of mental health issues, and greater severity of suicidality. However, this review did not have a primary focus on people who died by suicide. Furthermore, a systematic review by Hom et al. (9) examined barriers and facilitators of help-seeking for suicidality, but this review did not examine other factors associated with help-seeking, such as demographic and diagnostic characteristics.

We recognize that people may not use formal services for variety of reasons, including service inaccessibility or unaffordability, stigma, poor mental health literacy, preference for informal support, or negative past healthcare experiences. Identifying the characteristics associated with non-use of services within this population is critical to address these barriers, develop alternative pathways into services, re-design current services, and inform the development of novel interventions or more appropriate “care” models.

We focused on non-receipt of formal mental health services, rather than informal support as the primary outcome, given the focus of available literature, and since formal service use data is more easily verifiable by administrative health records linkage. Additionally, formal services are more likely to deliver evidence-based treatments compared to informal supports. One complexity in the literature is that studies consider a range of timeframes of mental health service use, ranging from non-use at time of death to non-use across an individual's lifetime. Shorter timeframe studies that classify decedents as “not in services” based on data from the weeks before death may miss service use in the months before death. Longer timeframe studies that classify decedents as “in services” based on lifetime use may inaccurately reflect service use in response to direct precipitants of suicide. In this review, we did not set limits on the timeframe examined; rather, this information was extracted from included studies. A second complexity is that studies vary in the data source used to ascertain service utilization and predictors of non-receipt of services. Studies that use psychological autopsies to acquire data through interviewing bereaved family, friends, or health professionals may yield unreliable reports (10). Studies that use coronial and/or health administrative data are also subject to methodological limitations (e.g., missing data) and are limited to examining risk factors that can be ascertained after death. To increase the comprehensiveness of the review, we included service use data and data on predictors of non-receipt of services from all aforementioned sources.

The aims of the review were to: (1) determine robust risk factors for non-receipt of formal mental health services among people who died by suicide, regardless of age; and (2) examine whether these risk factors are stable across different timeframes and types of services (e.g., inpatient vs. outpatient services).

## Materials and Methods

### Search Strategy and Selection Criteria

This systematic review adhered to PRISMA guidelines (11) and was prospectively registered with PROSPERO (CRD 42021226543). The research team included individuals with lived experience of suicidality, who helped to shape the inclusion criteria and interpretation of findings for this review. Four electronic databases (PsycInfo, PubMed, CINAHL, and Web of Science) were searched from 1^st^ January 1980 to 1st March 2021, with no language restrictions. The search strategy included a combination of three key blocks of terms related to: (i) suicide, (ii) health service utilization, and (iii) dying by suicide (see Supplementary Materials). Reference lists of included papers were examined to identify any additional papers.

Eligible studies were observational studies published in a peer-reviewed journal that examined one or more predictors of non-receipt of formal mental health services amongst people who died by suicide. Predictors specific to certain populations or regions (e.g., military rank, number of deployments, US census region, years spent living in a specific country) were excluded. Formal mental health services use was defined as seeing a general practitioner (GP), psychologist, psychiatrist, or other mental health professional in an emergency department (ED), inpatient or outpatient setting for mental health or substance use problems, or receiving a prescription for psychotropic medication. Studies that did not distinguish between mental health and non-mental health services, or formal and informal supports were excluded. No restrictions were placed on timeframe of service use, but the timeframe needed to be ascertainable from the article or through secondary sources (e.g., public information on the database). Studies that examined “past” or “previous” service contact were assumed to be examining lifetime service use.

Following removal of duplicates, all titles and abstracts were screened for suitability by ST, and a subset of 20% was screened by NR, with high interrater reliability (κ = 0.96). Both authors then independently screened full-text articles for inclusion (κ = 0.85). Any disagreements were resolved through discussion with a third author (HC). At the full-text screening stage, three non-English articles were translated into English using Google Translate. One article, in French, and another, in German, were manually cross-checked by AJM. The third article, in Norwegian, was corrected by checking unclear expressions using alternative translators or by exploring where unclear terms were used in other contexts.

### Data Analysis

ST and NR extracted data into a custom spreadsheet, recording: article, authors, year of publication, region/country, sample characteristics (size, age, and time period of death), data source(s), type of mental health service use (any, specialist, inpatient/ED, or mental health-related primary care), timeframe of mental health service use (within 1 month of death, within 1 year of death, or over 1 year prior to death/lifetime), predictors assessed, and main findings. Specialized mental health services include services provided within a particular setting (e.g., only outpatient, only a specified clinic/hospital) or by a particular type of mental health professional (e.g., only psychiatrists or psychologists).

A narrative synthesis approach was undertaken because significant variability in the designs, analyses and variables examined in included studies (see Supplementary Table 1) precluded a meta-analytic approach. Given the number and breadth of risk factors examined across studies, the below findings are reported by strength of evidence for an association with non-receipt of care, with variables with “consistent evidence” described first, followed by variables with “some evidence” and “unlikely association” for ease of interpretation of the results. See Table 1 for definitions.

**Table 1:** Description of strength of evidence ratings.

**Level of evidence**	**Description**
Consistent evidence	>50% of observations find a significant association in one direction, with no substantial significant findings in the opposite direction for variable is examined by ≥3 studies
Some evidence	30–50% of observations find a significant association in one direction, with no substantial significant findings in the opposite direction for variables examined by ≥3 studies
Unlikely association	<30% of observations found a significant association in one direction for variables examined by ≥3 studies
Insufficient studies	Variables examined by <3 studies

### Quality Assessment

Study quality was assessed using a modified version of the Joanna Briggs Institute Checklists for Cohort and Analytical Cross-Sectional Studies (12) (see Supplementary Table 2). Studies were rated “adequate,” “partial,” “poor,” or “unclear” on each item by two independent reviewers (ST and AA), with consensus achieved through discussion.

## Results

**Figure 1:**
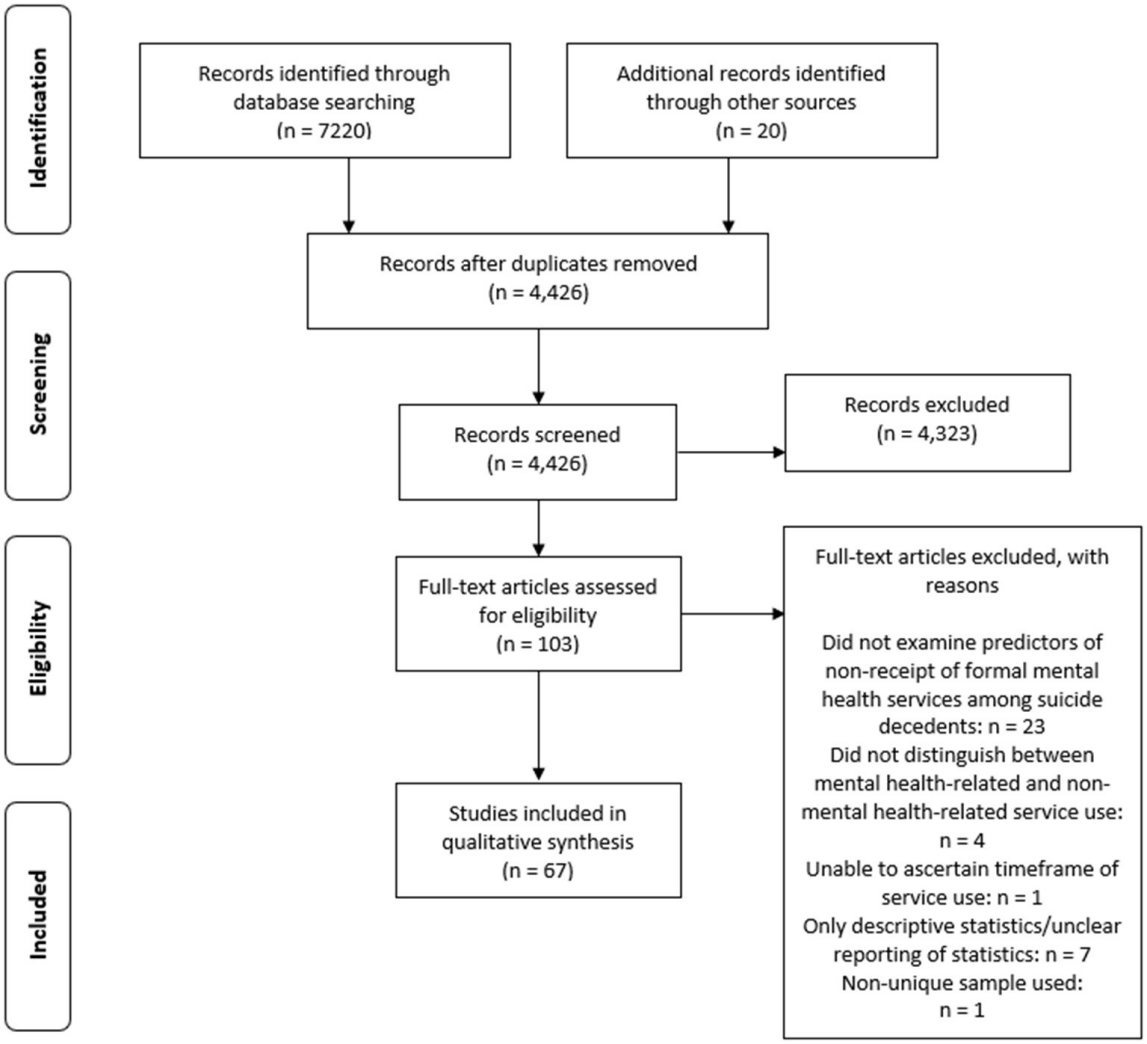
PRISMA flow diagram.

A total of 7,240 articles were identified. After removing duplicates and excluding studies based on abstracts, full text, or overlapping samples, 67 were eligible for inclusion (see Figure 1). The majority of included studies were from the United States (*n* = 19), the United Kingdom (*n* = 11), Canada (*n* = 8), Australia (*n* = 7), or Taiwan (*n* = 5). Most studies included a broad age range, though some exclusively examined adults over 50 (*n* = 8), or young people under 25 (*n* = 10). The type of services was highly varied, with “any” and “specialist outpatient” being most common. Timeframe of service use was also varied with lifetime, and within 1 year of death being most common (see Supplementary Table 1). Supplementary Table 3 presents characteristics of included studies. Table 2 presents a summary of the results for each risk factor. We examined findings across different timeframes and types of services and found little difference in the pattern of results, except for financial stressors where the association between experiencing a stressor and non-use of services was only seen for short timeframes of care (described below). As such, our results collapse across timeframe and type of mental health service use.

**Table 2:** Summary of variables assessed, including strength of evidence for the relationship between each variable and non-receipt of mental health services.

**Variable**	**Total # of studies**	**Total # of observations**	**Direction (variable associated with non-receipt of mental health services)**	**Total # of observations in this direction**	**Summary of evidence for risk factors for non-receipt of mental health services**
**Demographic**					
Sex	31	50	**Male**	**31**	**Consistent evidence for male sex**
			Null	19	
Age	23	30	**Older age**	**10**	Evidence for both younger and older age*
			**Younger age**	**8**	
			Middle age	1	
			Bimodal (older and younger age)	2	
			Null	9	
Location	8	14	**Rural location**	**8**	**Consistent evidence for rural location**
			Null	6	
Ethnicity	13	15	Non-White ethnicity	5	Some evidence for ethnic minority status
			Indigenous	2	
			Null	8	
Employment status	7	10	Being employed	4	Some evidence for being employed
			Being retired	1	
			Null	5	
Marital status	11	15	Being married	2	Unlikely association for marital status
			Never having been married	1	
			Being widowed	2	
			Null	10	
Living situation	6	7	Living alone	2	Unlikely association for living situation
			Null	5	
Education level	4	5	Low neighborhood education	2	Unlikely association for individual education level; insufficient evidence for neighborhood education level
			Null	3	
Occupation	7	9	Farm laborer (compared to farm manager)	1	Insufficient studies (given heterogeneity among studies)
			Non-farming/agriculture occupation among those living rurally	1	
			Marine Corp (compared to Army)	1	
			Null	6	
Income level	2	3	High income	2	Insufficient studies
			Low neighborhood income	1	
Religion	2	2	Null	2	Insufficient studies
Sexual orientation	1	2	Null	2	Insufficient studies
Parental status	1	2	No children	2	Insufficient studies
Perinatal status	1	5	Suicide death during perinatal period	1	Insufficient studies
			Null	4	
Political views	1	1	Being socially conservative	1	Insufficient studies
**Diagnostic**					
Any MH diagnosis	8	10	**Absence of any MH diagnosis**	**10**	**Consistent evidence for absence of any MH diagnosis**
Mood disorder/ depressed mood	10	15	**Absence of mood disorder/depressed mood**	**12**	**Consistent evidence for absence of mood disorder/depressed mood**
			Null	3	
Schizophrenia and related disorders	6	13*	**Absence of schizophrenia and related disorders**	**11**	**Consistent evidence for absence of schizophrenia and related disorders**
			Null	2	
Alcohol/substance abuse	10	15	**Absence of alcohol/substance abuse**	**8**	**Consistent evidence for absence of alcohol/substance use**
			Null	7	
Family history of mental illness/suicidality	4	6	Absence of family history of mental illness	1	Unlikely association for family history of mental illness/suicidality
Anxiety	2	3	Absence of anxiety disorder	2	Insufficient studies
			Null	1	
Personality disorder	2	2	Null	2	Insufficient studies
Adjustment disorder	1	2	Absence of adjustment disorder	2	Insufficient studies
Problem gambling	1	3	Absence of problem gambling	3	Insufficient studies
**Suicide-related**					
Method of suicide	20	24	**Violent methods**	**15**	**Consistent evidence for violent methods of suicide**
			Carbon monoxide	2	
			Null	7	
Past suicidal behavior	16	20	**Absence of past suicidal behavior**	**18**	**Consistent evidence for absence of past suicide attempts**
			Null	2	
Disclosure of suicide intent to others	7	9	Absence of disclosure	4	Some evidence for the absence of disclosure
			Null	5	
Location/time of death	4	4	Null	4	Unlikely association for location/time of death
Suicide note	7	7	No suicide note	2	Unlikely association for no suicide note
			Null	5	
Time from initial onset of suicidal behavior	1	2	Short time period between initial onset of suicidal behavior and death	2	Insufficient studies
Perceived suicide risk	2	2	Low perceived risk	1	Insufficient evidence
			Null	1	
**Psychosocial stressors/precipitating events**					
Any stressor	4	4	Presence of any recent stressor	2	Some evidence for presence of any stressor
			Null	2	
Financial/job problems	9	13	Presence of financial/job problems	4	Some evidence for presence of financial/job problems
			Null	9	
Criminal/legal problems	5	6	Presence of criminal/legal problems	2	Some evidence for experiencing criminal/legal problems
Physical health problems	10	11	Absence of physical health problems	1	Unlikely association for physical health problems
			Presence of physical health problems	3	
			Null	7	
Family or relationship or interpersonal problems	6	8	Presence of family/relationship/interpersonal problems	1	Unlikely association for experiencing family or relationship or interpersonal problems
			Null	7	
Recent loss (incl. suicide or other death of close person)	6	7	Null	7	Unlikely association for experiencing recent loss
Housing problems/homelessness	2	2	Being homeless	1	Insufficient studies
			Null	1	
Immigration	1	1	Null	1	Insufficient studies
Life events in childhood	1	1	Null	1	Insufficient studies
Perpetuating violence	1	1	Perpetrator of violence	1	Insufficient studies
Victim of violence	1	1	Null	1	Insufficient studies
**Other variables**					
Contact with other services (e.g., GP, social services)	6	10	**Absence of contact with other services**	**8**	**Consistent evidence for absence of contact with other services**
			Null	2	
Family history of psychiatric contact	1	2	Absence of family history of psychiatric contact	1	Insufficient studies
			Null	1	
Social problem-solving	1	1	Null	1	Insufficient studies
Impulsivity	1	1	Null	1	Insufficient studies
Academic performance	1	1	Null	1	Insufficient studies

### Consistent Evidence of Association With Non-receipt of Services

There was consistent evidence of an association between non-receipt of services and a number of demographic, diagnostic, and suicide-related variables. Most studies indicated that males were less likely to receive services compared to females (13–31). A number of studies found that male sex was only associated with non-receipt of services in certain subgroups [e.g., non-Indigenous Australians (32), young adults (33)] or under certain circumstances (34–36) [e.g., inpatient, not outpatient services (37), within 1 year of death, but not 1 month of death (34, 35)]. Although some studies found no relationship (24, 38–42), no study found that females were less likely to access services compared to males.

Age also predicted non-receipt of services in most studies. Some studies using general population samples (13, 21, 28, 43) and US military samples (24, 35) found that non-receipt of services was associated with younger age. In contrast, a number of studies of the general population found that non-receipt of services was associated with older age (18, 22, 30, 39). Almost all significant findings compared age groups using diverse age categories. Samples exclusively of young people under 25 (33), and of older adults over 65 had mixed findings (17, 44). While these findings may appear to be inconsistent, both young age and old age are likely risk factors for non-receipt of services. In line with this interpretation, two studies found that service use within 1 year of death was less pronounced among both younger *and* older decedents (20, 26). Six studies found no association (19, 38, 40, 45–47), which, when taken in the context of the other findings, may implicate a bimodal pattern of risk for both younger and older people, given that these studies included a broad age range and continuous age measures.

Regarding location, four studies found that decedents residing in rural locations were less likely to have received any mental health services compared to those residing in urban locations (27, 48–50). An Australian study found that non-receipt of mental health services was predicted by rural location only among Indigenous Australians (32). Three studies found no association (39, 44, 51).

Regarding diagnostic variables, studies consistently found that the absence of any mental health diagnosis predicted non-receipt of services across different timeframes and service types (38, 45, 46, 52–55). Specific diagnostic categories that were consistently associated with a greater likelihood of receiving services were mood disorders or symptoms, including depression and bipolar disorder (20, 26, 38, 44, 46, 56, 57), and schizophrenia and related disorders (37, 47, 58). There was also consistent evidence to suggest that non-receipt of services was associated with the absence of alcohol and/or substance abuse in general samples (37, 38, 50) and in certain subgroups [e.g., non-Indigenous Australians (32)]. An additional study found that non-receipt of services was associated with presence of blood alcohol at the time of death (59). However, four studies found no association for alcohol and/or substance abuse (44–46, 53).

Regarding suicide-related variables, the majority of studies found that non-receipt of mental health services was associated with more violent methods of suicide (e.g., firearm or hanging) compared to less violent methods (e.g., drug overdose) (13, 14, 20, 22, 25, 26, 39, 42, 50, 60–62). One study found that males, but not females, who were not receiving services were less likely to die by drug overdose compared to those receiving services (31). Two studies found that suicide by carbon monoxide poisoning was associated with not receiving services (50, 63). A minority of studies found no association (37, 38, 44, 45, 47, 64). All but two studies (47, 50) found that the absence of past suicidal behavior also consistently predicted non-receipt of services (14, 20, 25, 26, 31, 32, 37, 38, 42, 44–46, 64, 65). That is, decedents who had made a previous suicide attempt were more likely to have received services. Studies also consistently found that those not receiving non-mental health services, such as physical healthcare services and social services were also less likely to receive mental health services in the period before suicide (25, 31, 32, 45, 46, 49).

### Some Evidence of Association With Non-receipt of Services

There was some evidence of an association between non-receipt of services, and ethnicity and employment status. Several studies found that non-White compared to White (14, 20, 30, 35, 66), and Indigenous compared to non-Indigenous decedents (14, 20, 30, 66) were less likely to have received services. No studies found that ethnic minority groups were more likely to receive services, but a number found no association (19, 32). Among studies examining employment status, two found that non-receipt of mental health services was associated with being employed (47, 64), and one found that non-receipt of inpatient, but not outpatient services, was associated with being employed (37). In contrast, another study found that being retired, but not being employed, predicted lower odds of service use (39). Three studies found no association (42, 45, 46).

Regarding suicide-related variables, there was some evidence for an association between non-disclosure of suicidal intentions/ideation to others and non-receipt of mental health services. Some studies found that non-receipt of services was associated with the absence of disclosure in general samples (39, 67) and among certain subgroups (e.g., non-Indigenous Australians only) (32) and circumstances (e.g., for inpatient, but not outpatient service use) (37). However, three other studies found no relationship (45, 46, 50).

Regarding psychosocial stressors, some studies explored whether non-receipt of services was associated with experiencing any recent stressors. Two studies found that people who had not received services were more likely to have experienced a stressor prior to their deaths (26, 42), but another two found no such association (46, 64). Other studies examined the relationship between service use and a specific stressor, with some finding that non-receipt of mental health services in the year before death was associated with experiencing financial problems (20, 44, 47, 68), but not job problems (20, 44) at the time of death. Lifetime service use on the other hand, was not associated with financial or job problems (26, 45, 50, 69). This pattern suggests that recent experiences of financial problems may be a risk factor for dying by suicide without having received mental health services. Additionally, there was some, albeit limited, evidence to suggest that non-receipt of services was associated with experiencing criminal/legal problems prior to suicide (20, 26, 38, 44, 64).

### Unlikely Evidence of Association With Non-receipt of Services

The evidence suggested that there was an unlikely association between mental health service use and the following variables: marital status (50, 70, 71), individual education level (45, 47, 64), having a family history of mental illness/suicidality (25, 26, 35, 38, 39, 44–47, 50, 64), location or time of death (13, 45, 47, 64), the presence of a suicide note (37, 42, 45, 64), experiencing physical health problems (24, 44, 60, 72–75), family/relationship problems (25, 26, 39, 45), and experiencing a recent loss (14, 26, 37, 42, 45, 50). Most studies found no association between non-receipt of services and living situation (20, 26, 38, 44, 50, 64), although one study found that elderly decedents not receiving services were more likely to be living alone (25), which might suggest living alone confers a risk in older populations.

### Insufficient Evidence of Association With Non-receipt of Services

There was an insufficient number of studies examining all remaining variables (see Table 2). Although occupation was examined in a number of studies, these studies were highly heterogenous in the occupations examined and no specific occupation was examined by more than two studies (24, 44, 60, 72–75).

### Quality of Included Studies

Study quality assessment and ratings are presented in Supplementary Table 4. Most studies were adequate on Items 1 and 2, while close to half of studies were rated as partial for item 3 and poor for item 4. Only eight studies scored less than adequate on >50% of the items (31, 36, 53, 54, 56, 68, 73, 76). Excluding these studies did not meaningfully alter the results.

## Discussion

Key factors associated with non-receipt of formal mental health services before suicide death identified in this review were male sex, younger or older age, and rural location. These findings are largely consistent with previous reviews of service use among people with mental health issues (6, 7). People not receiving services were less likely to have a psychiatric diagnosis, or contact with non-mental health services. They were also less likely to have past suicidal behavior and more likely to use violent means of suicide, which might suggest their first suicide attempt tends to be fatal. Mood and schizophrenia spectrum disorders were associated with greater likelihood of service receipt compared to other diagnoses. There was some evidence to suggest that people who died by suicide without having received services were more likely to be from an ethnic minority background, and have experienced stressors, such as financial problems.

Consistent with previous research showing that men are less likely to use all types of health services (77), we found that males were less likely than females to have received formal mental health services prior to suicide. This disparity may be due to men's higher levels of stigma toward mental illness and help-seeking (78–82) and/or lack of services that align with men's preferences (83). There is also evidence of lower levels of mental health literacy among men, meaning they may be less likely to recognize symptoms, and less aware of where or how to access help (82). Findings related to the association between non-receipt of services and more violent means of death and the absence of suicide attempt may also be mediated by sex, in that women are more likely to attempt suicide compared to men (84), while men are more likely to use more violent suicide means (85). The use of less violent methods among women may result in higher survival rates for attempts, and subsequent linkage to healthcare services. Lower rates of mental health service utilization among men may, at least partly, contribute to higher global suicide rates in men relative to women (86), indicating that future research should focus on developing and evaluating service pathways tailored to men's needs.

The association between rural location and non-receipt of clinical services may be due to a number of factors, including geographic and structural barriers to accessing services (87), a culture of self-reliance among those living rurally (88), and threats to livelihood like physical injury and drought (89). There was also some evidence that experiencing financial problems and ethnic minority status were associated with reduced likelihood of service use. Each of these variables are associated with increased risk of suicide (90–93) and highlight the impact of cultural factors and social determinants of health on service use (94).

Decedents with a psychiatric diagnosis were more likely to have received services compared to those without a diagnosis, which likely reflects that acquiring a formal diagnosis necessarily requires contact with a professional. In contrast, suicide among people without diagnoses might be more likely to have been precipitated by acute stressors, rather than enduring mental health issues, providing little opportunity for intervention. Indeed, we found some, albeit mixed, evidence to suggest that those experiencing acute stressors, such as financial difficulties, prior to suicide were less likely to have received services compared to those not experiencing such stressors.

It is possible that there are distinct profiles among those not receiving mental health services. For instance, we found evidence that both younger and older age were associated with non-receipt of services, potentially reflecting different subgroups. Younger individuals are more likely to report financial concerns and fear of psychotherapy as barriers to help-seeking, whereas elderly people may be less likely to receive formal mental health services due to difficulties with transportation and/or a tendency to normalize distress (95). We also found some inconsistent evidence for the relationship between service use, and ethnicity and employment status, which might further indicate that people who die by suicide without having received services have diverse profiles.

One limitation of the current review was that all included studies were conducted in high-income countries, meaning the findings may not apply to low and middle-income countries, where 75.5% of global suicides occur (96). Included studies also varied in the range of covariates accounted for, which may impact the significance and comparability of results. Furthermore, we assessed the reliability and validity, but not the comprehensiveness of the service utilization data. Studies varied in the breadth of healthcare utilization data that they captured, with many studies effectively capturing public but not private mental health service utilization. Although accessing data on all possible sources of mental health service use may not be practicable in many cases, mental health service use is likely to be underreported within databases, impacting the accuracy with which studies could determine service use. However, despite variation in the quality of included studies, key findings in relation to demographic, diagnostic, suicide-related, and service contact variables were largely consistent across studies. Additionally, the use of coronial and similar data also limited the type of predictors that could be examined. Few studies examined such variables as sexual orientation and personality characteristics, while no studies examined the presence of adverse childhood experiences (ACEs), despite the well-established association between ACEs, and both suicidality and self-harm (97, 98). Further, given the current focus on individuals who died by suicide, we were unable to gain insight into service preferences and barriers to service use in this population. Future research should examine these characteristics among individuals at risk of suicide to help guide clinical practice and inform the development of alternative service pathways.

The current review was also unable to distinguish between people who chose not to seek help and people who attempted to access help without successfully receiving it. These subgroups are likely to have different needs and barriers to service access, highlighting an important avenue for future research. Moreover, this review only examined predictors of non-receipt of *formal* mental health services. These services may not always meet users' needs and may vary in quality (99). Receiving formal services also does not guarantee that individuals were recognized as suicidal or treated for suicidality. The quality of services received within the formal mental health system also varies considerably and may not always meet the individual needs of those within this system (99). Furthermore, those at risk of suicide—particularly those in younger age groups—also often rely on informal sources of support, such as support from family and friends, and crisis lines or internet forums. The latter sources remove additional barriers to help-seeking such as cost and confidentiality (100, 101). These informal supports may be helpful, or indeed sufficient, for some people at risk of suicide (102). For example, calls to suicide crisis lines are a form of help-seeking not captured in this review that may be effective in alleviating short-term distress for some people (102). Understanding the characteristics of suicidal individuals who do not receive either formal or informal sources of support is necessary to identify those least likely to receive any form of help.

Further work is needed to understand the trajectories and antecedents to suicide, and barriers to using mental health services to aid the development of appropriate interventions. Epidemiological studies provide broad data on risk factors. However, within-group patterns and individual trajectories may be more clearly elucidated through surveys of those “at risk” outside of services and qualitative interviews with people in our target group that have made a non-fatal attempt, as well as families and friends bereaved by suicide. By providing insight into the individual stories and needs of people at risk of suicide, these approaches will facilitate the development of tailored and preference-based service pathways (103).

Our conclusion from this review is that people who die by suicide without receiving formal mental health services are likely to have diverse characteristics. It is likely that a multi-faceted approach may increase engagement. Clearly, services that are currently on offer do not attract or reach this group. New pathways, such as alternatives to the ED (e.g., peer support services, safe havens), and outreach activities, potentially integrated in non-health settings such as workplaces, welfare and housing agencies, and educational institutions, are needed (see Box 1 for research implications). Online interventions, which have been shown to reduce suicidal ideation and reduce barriers such as high out-of-pocket expenses, physical distance and stigma, may also offer a route into services (104). However, we do not know whether the above avenues will work, and more investigation is needed. Furthermore, significant reform is likely needed to provide timely support to those experiencing an acute stressor given that existing services are subject to long waitlists, and ED responses are often perceived as unsatisfactory (105). Further, people experiencing suicidal distress may not recognize the role of mental health support in managing acute distress. Our findings on the relationship between social determinants of mental health and mental health service use among people who die by suicide suggest that equity issues and broader policy reform in relation to such issues as social welfare, employment, education, and housing should be considered.

Box 1 **Implications of research findings.**

Reaching people who are “under the radar” before they die by suicide may require:

- Reducing inequity of access to existing services including financial and locational barriers
- Trialing outreach approaches and interventions that are designed to reach people in the short interval between experiencing a life stressor (e.g., financial adversity) and suicide
- Providing alternative pathways into services including capitalizing on existing government service touchpoints (e.g., income support, social housing, transition periods)
- Implementing and evaluating alternative forms of support including safe havens, respite services, and the peer support workforce
- Offering and evaluating online support and referral services for suicidality

Due to diversity among people who die by suicide without receiving services, there are likely several avenues for increasing the service use for those experiencing suicidality, including improving access, quality, and delivery of existing services and the development of new support pathways. Further research exploring reasons underlying non-use of existing services is critical to better meet the needs of people at risk of suicide.

## Data Availability Statement

The original contributions presented in the study are included in the article/Supplementary Material, further inquiries can be directed to the corresponding author/s.

## Author Contributions

ST, NR, PB, AC, GC, AM, and HC designed the study. ST and NR conducted the literature search, screened titles, abstracts, and full-texts for eligibility for inclusion into the review, and extracted data from the manuscripts. ST and AA performed quality assessments. AM translated non-English articles to English. ST, NR, and AA wrote the first draft of the manuscript. All authors contributed to the interpretation and subsequent edits of the manuscript.

## Funding

Funding for this research was provided by the Medical Research Future Fund, Australia (grant number: 1200195). PB, AC, and HC are supported by National Health and Medical Research Council Fellowships 1158707, 1173146, and 1155614.

## Conflict of Interest

The authors declare that the research was conducted in the absence of any commercial or financial relationships that could be construed as a potential conflict of interest.

## Publisher's Note

All claims expressed in this article are solely those of the authors and do not necessarily represent those of their affiliated organizations, or those of the publisher, the editors and the reviewers. Any product that may be evaluated in this article, or claim that may be made by its manufacturer, is not guaranteed or endorsed by the publisher.
