# Corrigendum: People who die by suicide without receiving mental health services: a systematic review

**DOI:** 10.3389/fpubh.2025.1623622

**Published:** 2025-06-27

**Authors:** Samantha Tang, Natalie M. Reily, Andrew F. Arena, Philip J. Batterham, Alison L. Calear, Gregory L. Carter, Andrew J. Mackinnon, Helen Christensen

**Affiliations:** ^1^Black Dog Institute, University of New South Wales, Sydney, NSW, Australia; ^2^Centre for Mental Health Research, Research School of Population Health, Australian National University, Canberra, ACT, Australia; ^3^School of Medicine and Public Health, University of Newcastle, Newcastle, NSW, Australia

**Keywords:** suicide, systematic review, healthcare utilization, coronial data, mental health services

In the published article, there was an error made in the Abstract and Introduction regarding the prevalence of mental health service use prior to suicide. In the Abstract the sentence previously stated that:

“The majority of people who die by suicide have never seen a mental health professional or been diagnosed with a mental illness.”

The corrected sentence appears below:

“The majority of people who die by suicide have not had contact with mental health professionals in the year prior to death.”

Further, in the first paragraph of the Introduction the sentence previously stated:

“Notably, over two-thirds of individuals who die from suicide do not receive professional mental health support.”

The corrected sentence appears below:

“Notably, over two-thirds of individuals who die from suicide do not receive professional mental health support (including seeing a general practitioner or mental health professional for mental health or substance use reasons) in the year prior to death.”

The original article has been updated.

