# Supplementary material for: People Who Die by Suicide Without Receiving Mental Health Services: A Systematic Review
Source: Front Public Health. 2022 Jan 18;9:736948. doi: 10.3389/fpubh.2021.736948 (PMC8804173; doi:10.3389/fpubh.2021.736948)
Supplement: Supplementary file 1 [file Table_1.docx]

**Supplementary material: PsycINFO search strategy**

1. (suicid* or "self poison*" or "self-poison*").mp. [mp=title, abstract, heading word, table of contents, key concepts, original title, tests & measures, mesh]

2. ("mental health treatment*" or "mental health service*" or "mental health contact*" or "health service**" or "healthcare*" or "health care*" or "helpseek*" or "help seek*" or "help-seek*" or "service use*" or "service utili?ation").mp. [mp=title, abstract, heading word, table of contents, key concepts, original title, tests & measures, mesh]

3. ("decedent*" or "death*" or "deceased" or "coroner*" or "coronial" or "retrospective" or "suicide case*" or "die* by suicide").mp. [mp=title, abstract, heading word, table of contents, key concepts, original title, tests & measures, mesh]

4. 1 and 2 and 3

5. limit 4 to yr="1980–Current"

**Supplementary Table 1. Timeframe and type of mental health service use examined by each included study.**

| **Study** | **≤1 month, non-receipt of:** | | | | **>1 month≤1 year, non-receipt of:** | | | | **>1 year, non-receipt of:** | | | |  |
| --- | --- | --- | --- | --- | --- | --- | --- | --- | --- | --- | --- | --- | --- |
|  | **Overall MH services (%)** | **Specialist MH services (%)** | **Inpatient/ED MH services (%)** | **MH-related primary care services (%)** | **Overall MH services (%)** | **Specialist MH services (%)** | **Inpatient/ED MH services (%)** | **MH-related primary care services (%)** | **Overall MH services (%)** | **Specialist MH services (%)** | **Inpatient/ED MH services (%)** | **MH-related primary care services (%)** |  |
| Ahmedani, Simon (2014) | • |  |  |  | • |  |  |  |  |  |  |  |  |
| Ali, Rockett (2021) |  |  |  |  | • |  |  |  |  |  |  |  |  |
| Arnautovska, McPhedran (2015) |  |  |  |  | • |  |  |  |  |  |  |  |  |
| Bakst, Braun (2014) | • |  |  |  |  |  |  |  | • |  |  |  |  |
| Betz, Krzyzaniak (2011) |  |  |  |  | • |  |  |  |  |  |  |  |  |
| Booth, Briscoe (2000) |  |  |  |  |  |  |  |  | • | • | • |  |  |
| Chang, Liao (2009) |  |  |  |  |  | • |  |  |  |  |  |  |  |
| Chang, Lai (2012) |  | • | • |  |  |  |  |  |  |  |  |  |  |
| Chen, Liao (2009) |  |  |  |  |  | • |  |  |  |  |  |  |  |
| Cheung, Douwes (2017) |  |  |  |  |  |  |  |  | • |  |  |  |  |
| Cho, Kang (2013) |  |  |  |  | • |  |  |  |  |  |  |  |  |
| Choi, DiNitto (2017) |  |  |  |  | • |  |  |  |  |  |  |  |  |
| Choi, DiNitto, Marti, Conwell (2019) |  |  |  |  | • |  |  |  |  |  |  |  |  |
| Choi, DiNitto, Marti (2019) |  |  |  |  | • |  |  |  |  |  |  |  |  |
| Coope, Donovan (2015) |  |  |  |  |  |  |  |  |  | • |  |  |  |
| De Leo, Draper (2013) |  |  |  |  |  | • |  |  |  |  |  |  |  |
| Foster, Gillespie (1997) |  |  |  |  |  |  |  |  | • |  |  |  |  |
| Frei, Bucher (2013) |  |  |  |  |  |  |  |  |  | • |  |  |  |
| Giupponi, Pycha (2014) |  |  |  |  |  |  |  |  | • |  |  |  |  |
| Grigoriadis, Wilton (2017) | • |  |  | • | • | • |  | • |  |  |  |  |  |
| Haaland, Bjørkhold (2017) |  |  |  |  | • |  |  |  |  |  |  |  |  |
| Hamdi, Price (2008) |  |  |  |  | • |  |  |  |  |  |  |  |  |
| Hayward, Zubrick (1992) |  |  |  |  |  |  |  |  | • |  |  |  | |
| Hintikka, Lehtonen (1997) |  |  |  |  |  | • |  |  |  |  |  |  | |
| Ho, Ho (2014) |  |  |  |  |  |  |  |  |  | • |  |  |  |
| Isometsa, Henriksson (1994) |  |  |  |  |  | • | • |  |  | • | • |  |  |
| Joe, Marcus (2007) |  |  |  |  | • |  |  |  |  |  |  |  | |
| Jung, Lee (2019) |  |  |  |  |  |  |  |  | • | • |  |  |  |
| Kameyama, Matsumoto (2011) |  |  |  |  | • |  |  |  |  |  |  |  |  |
| Kavalidou, McPhedran ) (2015) |  |  |  |  | • |  |  |  | • |  |  |  |  |
| King (2001) |  |  |  |  |  | • |  |  |  |  |  |  | |
| King, Schlichthorst (2020) |  |  |  |  |  | • |  | • |  |  |  |  |  |
| Law, Wong (2010) |  |  |  |  |  | • |  |  |  |  |  |  |  |
| Law, Wong (2015) |  |  |  |  |  |  |  |  | • |  |  |  |  |
| Lee, Lin (2008) | • |  |  |  | • |  |  |  |  |  |  |  |  |
| Loh, Tai (2012) |  |  |  |  |  |  |  |  | • |  |  |  | |
| Malchy, Enns (1997) |  |  |  |  |  |  |  |  |  | • |  |  |  |
| McLone, Kouvelis (2016) |  |  |  |  | • |  |  |  |  |  |  |  |  |
| McPhedran and De Leo (2013) |  |  |  |  | • |  |  |  |  |  |  |  |  |
| Niederkrotenthaler, Logan (2014) |  |  |  |  | • |  |  |  |  |  |  |  |  |
| Pennington, Ylitalo (2021) |  |  |  |  |  |  |  |  | • |  |  |  |  |
| Persons, Hefti (2019) |  |  |  |  |  |  |  |  | • |  |  |  |  |
| Pitkälä, Isometsä (2000) |  |  |  |  |  |  |  |  |  | • |  |  |  |
| Renaud, Berlim (2009) | • | • |  |  | • | • |  |  |  |  |  |  |  |
| Renaud, Berlim (2010) |  | • |  |  |  | • |  |  |  |  |  |  |  |
| Ribeiro, Gutierrez (2017) | • |  |  |  | • |  |  |  |  |  |  |  |  |
| Roberts (2019) |  |  |  |  | • |  |  |  |  |  |  |  |  |
| Rodway, Tham (2020) |  |  |  |  | • |  |  |  |  |  |  |  |  |
| Runeson (1992) |  |  |  |  |  |  |  |  |  | • | • |  | |
| Ryan, Ghahramanlou-Holloway (2020) |  |  |  |  |  |  |  |  | • |  |  |  |  |
| Salib and Green (2003) |  |  |  |  |  |  |  |  |  | • |  |  |  |
| Schaffer, Sinyor (2016) |  |  |  |  | • |  |  |  |  |  |  |  |  |
| Schaffer, Sinyor (2014) | • |  |  |  |  |  |  |  |  |  |  |  |  |
| Schmutte and Wilkinson (2020) |  |  |  |  |  |  |  |  | • |  |  |  |  |
| Searles, Valley (2014) |  |  |  |  | • |  |  |  | • |  |  |  |  |
| Séguin, Boyer (2010) | • |  |  |  | • |  |  |  | • |  |  |  |  |
| Shahpesandy, Oakes (2014) |  |  |  |  |  |  |  |  | • |  |  |  |  |
| Stark, Huc (2012) | • |  |  |  | • |  |  |  | • |  |  |  |  |
| Sundqvist‐Stensman (1987) |  |  |  |  |  |  |  |  |  | • |  |  |  |
| Suso-Ribera, Mora-Marín (2018) |  |  |  |  |  | • |  | • |  |  | • |  | |
| Sveticic, Milner (2012) |  |  |  |  | • |  |  |  | • |  |  |  |  |
| Sweeney, Fontanella (2020) |  |  |  |  | • |  |  |  |  |  |  |  |  |
| Vassilas and Morgan (1997) |  |  |  |  | • |  |  |  |  | • |  |  |  |
| Waitz‐Kudla, Daruwala (2019) |  |  |  |  |  |  |  |  | • |  |  |  |  |
| Windfuhr, While (2008) |  |  |  |  | • |  |  |  |  |  |  |  |  |
| Wong, Wang (2017) |  |  |  |  | • |  |  |  |  |  |  |  |  |
| Zaheer, Jacob (2018) | • | • | • | • |  |  |  |  | • | • | • | • |  |

Note: Specialised MH services include services provided within a particular setting (e.g., only outpatient, only a specified clinic/hospital) or by a particular type of MH professional (e.g., only Psychiatrists).

**Supplementary Table 2. Quality assessment tool and ratings of all included studies**

**Quality assessment tool.**

| **1.** **Were objective, standard criteria used for measurement of the condition (dying by suicide)?** | |
| --- | --- |
| Adequate | Cases were defined by a coronial verdict of suicide. Open verdicts may be included only if they were screened to ensure suicide was probable (the most likely cause of death), rather than just possible (where there remains a large possibility of other causes). |
| Partial | Cases included verdicts of suicide and all open verdicts. |
| Poor | Studies that relied on unconfirmed or informant reports of suicide. |
| Unclear | Insufficient description provided. |
| **2.** **Was the exposure (i.e., risk factors) measured in a valid and reliable way?** | |
| Adequate | Use of official records (e.g., coronial files, health administrative databases), and/or the risk factors assessed purely through psychological autopsy methods are not prone to bias (e.g., demographic variables only). |
| Partial | Some measured risk factors are prone to bias (e.g., purely psychological autopsy methods used to measure psychosocial stressors including childhood trauma, financial strain, etc) and others are not. |
| Poor | All risk factors are prone to bias as described above. |
| Unclear | Insufficient description of the measurement of risk factors provided. |
| **3.** **Was the outcome (i.e., non-receipt of formal mental health services) measured in a valid and reliable way?** | |
| Adequate | Formal mental health service use was established through data linkage of official service databases (e.g. national health databases, hospital records). |
| Partial | Study used a combination of linked data from official service databases and informant reports from psychological autopsies (i.e. the source of information for some participants may have come purely from psychological autopsies). |
| Poor | Study solely used informant reports from psychological autopsies. |
| Unclear | Insufficient description provided (e.g., source of mental health care data is unclear). |
| **4.** **Were appropriate confounding factors accounted for?** | |
| Adequate | Appropriate confounding variables were accounted for within the study design or data analysis concerning mental health service utilisation (e.g., by matching or stratifying sampling of participants or by covariate adjustment in the data analysis). |
| Partial | Confounding variables were accounted for in the study design/analysis, but the chosen variables are subject to criticism due to having considerable conceptual/empirical overlap. |
| Poor | Confounding variables were not accounted for within the study design/analysis regarding mental health service utilisation. |
| Unclear | It is unclear whether/how confounding variables were accounted for. |

**Supplementary Table 3. Characteristics of included studies.**

| **Author (year)** | **Region, country** | **Sample size** | **Age** | **Included cases (data source)** | **Data source(s) for predictors and MHS use*** | **Type and timeframe of MHS examined** | **Predictors assessed** |  |
| --- | --- | --- | --- | --- | --- | --- | --- | --- |
| Ahmedani, Simon (2014) | United States | 5894 | All ages (range and mean not provided) | Health plan member suicides from 2000–2010 identified by ICD-10 codes X60–X84 (MH Research Network). | Electronic medical records and insurance claim data held by MH Research Network health systems | MHS visits within 4 weeks and 12 months before death. | Sex; age; neighbourhood income; neighbourhood education; method of suicide |  |
| Ali, Rockett (2021) | United States | 7038 | 10-19 (M=16.74, SD=2.09) | Youth suicides from 2006-2015 (NVDRS). | NVDRS | MH/substance abuse treatment, including pharmacotherapy and counselling, within 2 months before death. | Sex; ethnicity; past suicide attempts; method of suicide |  |
| Arnautovska, McPhedran (2015) | Queensland, Australia | Total: 147  Farm managers: 78  Farm labourers: 69 | All ages (range and mean not provided) | Farm managers and farm labourers who died by suicide from 2000–2009 (QSR and National Coronial Information System). Cases were classified as suicide ‘beyond reasonable doubt’ or ‘probable’ according to QSR. | QSR | Contact with a MH professional within 3 months before death. | Occupation |  |
| Bakst, Braun (2014) | Tel Aviv, Israel | Total: 98  Psychiatric diagnosis: 33  No psychiatric diagnosis: 65 | All ages (range and mean not provided) | All suicides from 2007–2010 (Tel Aviv death certificates) | Predictors: Israeli Psychiatric Central Register  MHS use: interviews with first-degree family members | Contact with MH practitioners (including psychologist, psychiatrist, social worker or community service worker) or receipt of pharmacotherapy over lifetime and within 4 weeks before death. | Any MH diagnosis |  |
| Betz, Krzyzaniak (2011) | Colorado, United States | Total: 3804  Hispanic: 434  Non-Hispanic White: 3370 | All ages (range not provided)  Hispanic: M=34.8, SD=14.4  Non-Hispanic White: M=46.1, SD=17.4 | All suicides from 2004–2008 (COVDRS) | COVDRS | MH/substance abuse treatment, including pharmacotherapy and counselling, within 2 months before death. | Ethnicity |  |
| Booth, Briscoe (2000) | Exeter Health District, England | Total: 126  Farmers: 63  Non-farmers: 63 | 16+  Farmers: M=53.8  Non-farmers: M=54.6 | Male farmers and non-farmers whose deaths were classified as ‘suicide’ or ‘open verdict’ from 1979–1994 (Exeter Health District database). | Coroner's inquest files; GP notes; psychiatric and community MH team records | Treatment as an inpatient or day patient at time of death; previous inpatient treatment; current or previous contact with outpatient or community MH teams; contact with MHS over lifetime; prescription of antidepressants before death. | Occupation |  |
| Chang, Liao (2009) | Taiwan | 12497 | All ages  (M=49.2, SD=18) | All suicides from 2001–2004 identified by ICD-9 codes E950–E959 (NHID). | NHID | Contact with a psychiatrist within 12 months before death. | Sex |  |
| Chang, Lai (2012) | Taiwan | 862 | 12–25 | Youth suicides from 2001-2004 identified by ICD-9 codes E950-E959 (Department of Health mortality database). | NHID | Contact with psychiatric inpatient and outpatient services within 30 days before death. | Sex; age |  |
| Chen, Liao (2009) | Taiwan | 2262 | Not specified | All suicides from 2000–2004 identified by ICD-9 code E952, E953, or E950 (Taiwanese mortality data). | NHID | Consultation with a psychiatrist within 12 months before death. | Method of suicide |  |
| Cheung, Douwes (2017) | New Zealand | Total: 214  Terminal cancer: 23  Non-terminal cancer: 191 | 65+ | Older suicides from July 2007–December 2012 (New Zealand Coronial Services). | New Zealand Coronial Services | Previous contact with MHS; previous psychiatric admissions. | Physical health problem (terminal cancer) |  |
| Cho, Kang (2013) | South Korea | 11523 | All ages (range and mean not provided) | All suicides from 2004 (Death Statistics Database of the Korean National Statistical Office and post-mortem examination findings provided by the Korean National Police Agency). | Predictors: Death Statistics Database and Korean National Police Agency  MHS use: Health Insurance Review and Assessment Service | Medical services contact in relation to psychiatric disorders, identified through ICD codes F00-99, within 12 months before death. | Sex |  |
| Choi, DiNitto (2017) | United States | Total: 43857  Non-disclosers: 35886  Disclosers: 10971 | 50+ | Older suicides from 2005–2014 (NVDRS). | NVDRS | MH/substance abuse treatment, including pharmacotherapy and counselling, within 2 months before death. | Disclosure of suicide intent |  |
| Choi, DiNitto, Marti, Conwell (2019) | United States | Total: 16924  Suicide not precipitated by physical health problem: 8389  Suicide precipitated by physical health problem: 8535 | 65+ | Older suicides from 2005–2014 (NVDRS). | NVDRS | MH/substance abuse treatment, including pharmacotherapy and counselling, within 2 months before death. | Physical health problem |  |
| Choi, DiNitto, Marti (2019) | United States | 1727 | 14-82  (M=36.1, SD=11.3) | People who died by suicide while in custody from 2005–2014 (NVDRS). | NVDRS | MH/substance abuse treatment, including pharmacotherapy and counselling, within 2 months before death. | Sex; age; ethnicity; marital status; any MH diagnosis; depression; alcohol/substance use; past suicidal behaviour; method of suicide; physical health problem; relationship/family problems; perpetrating violence; recent loss |  |
| Coope, Donovan (2015) | England, United Kingdom | 286 | 16–65  Not at all–some recession-related: M=41.3, SD=12.4  A lot–completely recession-related: M=45.5 SD=11.2 | Working aged suicides from 2010–2011 (Coroner's ledgers and databases from Avon, Hampshire, Manchester and Northampton). Excluded deaths given narrative or open verdicts. | Predictors and MHS use: coronial records.  Predictors: researchers’ ratings of the degree to which suicides were related to the recession, focusing on information concerning employment and financial problems. | Lifetime contact with psychiatric services. | Financial/job problems (i.e., degree to which suicide is recession-related) |  |
| De Leo, Draper (2013) | Queensland and New South Wales, Australia | 261 | 35+ | Suicides whose next of kin and health professionals were interviewed from 2006–2008 (Queensland Office of the State Coroner; Queensland Police Service; Glebe Coroner's Court). Time period of deaths not specified. | Predictors and MHS use: interviews with next of kin.  MHS use: retrospective psychiatric assessments by 2 psychiatrists. | Contact with psychiatrists within 3 months before death. | Any MH diagnosis; depression; anxiety; schizophrenia and related disorders; alcohol/substance use |  |
| Foster, Gillespie (1997) | Northern Ireland, United Kingdom | 118 | 14+ | Suspected suicides between 20 July 1992 and 19 July 1993 screened by author (Northern Ireland coronial records). 129/154 of identified suicides were later officially classified as suicides. | Predictors: coronial records.  MHS use: interview with GP; interviews with deceased's family members or close friends. | Lifetime contact with MHS. | Age |  |
| Frei, Bucher (2013) | Lucerne, Switzerland | 232 | All ages (range and mean not provided) | All suicides or probable suicides identified by ICD-10 codes X60-X84 from 2002–2006 (Swiss mortality statistics). Excluded assisted suicides. | Predictors: Swiss mortality statistics  Predictors and MHS use: coronial files; archives of the cantonal psychiatric institutions | Registration as a patient in a public psychiatric institution within 3 years before death. | Sex; age; employment status; marital status; location; occupation; religion; disclosure of suicide intent; method of suicide; presence of suicide note; location of death; physical health problem |  |
| Giupponi, Pycha (2014) | South Tirol, Italy | 396 | 12–94 (Mean = 52.69, SD = 18.02) | Suicides without illicit drug use disorders from 1997–2007 (Provincial Departments of Public Health death certificates). Excluded suicides where researchers were unable to ascertain whether subject was known to MHS. | Predictors: Provincial Departments of Public Health data; registered-persons database (MENTA)  Predictors and MHS use: interviews with informants (including family, partners, friends). | Contact with a MH professional (including psychologist or psychiatrist) working in the public health service before death. | Sex; age; ethnicity; employment status; marital status; education level; living situation; any MH diagnosis; alcohol/substance use; past suicidal behaviour; disclosure of suicide intent; method of suicide; presence of suicide note; location of death; family history of mental illness; family history of suicidal behaviours; financial/job problems; relationship/family problems; recent loss; life events in childhood; contact with GP |  |
| Grigoriadis, Wilton (2017) | Ontario, Canada | Total: 1648  Perinatal: 51  Non-perinatal: 1597 | 18–45 | Female suicides from 1994–2008 (OCCO) | Coronial records or health administrative data within the ICES. | Outpatient MH contact (including contact with a psychiatrist or a MH visit with a primary care provider), within 30 days and 12 months before death. | Perinatal status |  |
| Haaland, Bjørkhold (2017) | Agder counties, Norway | 329 | All ages (range and mean not provided) | All suicides from 2004–2013 (Cause of Death Register). | Predictors: Cause of Death Register.  MHS use: Sørlandet Hospital electronic medical records. | Contact with MHS and/or interdisciplinary specialised drug treatment within 12 months before death. | Sex; age |  |
| Hamdi, Price (2008) | West Kent, United Kingdom | Total: 479  MHS contact: 138  No MHS contact: 341 | 15–93 | All suicides from 2000–2004 (Kent County Coroner’s records). Included open verdicts and 14 verdicts of death by accident or misadventure screened as probable suicide. | Coronial records; GP records; questionnaire completed by relevant GP | Contact with MHS within 12 months before death. | Sex; age; ethnicity; employment status; marital status; living situation; MH diagnosis; depression; anxiety; schizophrenia and related disorder; personality disorder; alcohol/substance use; past suicidal behaviour; perceived suicide risk; disclosure of suicide intent; recent stressor; physical health problem; contact with GP; contact with social services; awaiting MH assessment at time of death |  |
| Hayward, Zubrick (1992) | Western Australia, Australia | 515 | M=43.3, SD=17.5 | All suicides from 1986–1988 (coronial records). | Coronial records | Help-seeking from health professionals (including GPs and psychiatrists) prior to suicide. | Alcohol consumption prior to death |  |
| Hintikka, Lehtonen (1997) | Kuopio province, Eastern Finland | 68 | 15-24 | Male youth suicides from 1988–1995 (death certificates) | Predictors: Police and forensic examinations of causes of death  MHS use: Medicolegal investigations conducted as part of Finnish National Suicide Prevention Project | Psychiatric consultations and treatments within 3 months before death. | Method of suicide | |
| Ho, Ho (2014) | Singapore | Total: 409  History of suicidal behaviour: 65  No history of suicidal behaviour: 344 | 60+ (M=73.6, SD=9.5) | Older suicides from 2000–2004 (Singapore Registry of Birth and Death coronial records). | Coronial records | Previous psychiatric treatment; previous admission to a mental hospital under MH legislation. | Previous suicidal behaviour |  |
| Isometsa, Henriksson (1994) | Finland | Total: 102  Bipolar Disorder: 31  MDD: 71 | All ages (range and mean not provided) | All suicides between 1^st^ April 1987 and 31^st^ March 1998 (autopsy and forensic examinations). | Predictors and MHS use: interviews with relatives and attending healthcare personnel; psychiatric, medical and social agency records; suicide notes  Predictors: retrospective psychiatric assessments by 2–3 psychiatrists. | Psychiatric treatment over lifetime and 12 months before death; psychiatric hospitalisation over lifetime and 12 months before death. | Bipolar Disorder |  |
| Joe, Marcus (2007) | United States | 1616 | 15+ | Suicides from 1993 identified by ICD codes E950.0–E958.9 (death certificates within the 1993 Current Mortality Sample). | Predictor: ICD codes listed on death certificates.  MHS use: interviews with next-of-kin. | Contact with a mental health professional during the 12 months before death | Method of suicide |  |
| Jung, Lee (2019) | South Korea | Total: 284  Male: 168  Female: 116 | Males: M=15.98, SD=1.47  Females: M=15.75, SD=1.45 | Middle and high school student suicides from 2014–2016 (Ministry of Education). | Ministry of Education student suicide reports. | Previous receipt of counselling sessions at school; previous receipt of professional help, including visiting an external counselling centre or mental hospital. | Sex |  |
| Kameyama, Matsumoto (2011) | Japan | Total: 39  Debt: 16  No debt: 23 | 30–64 (M=44.9, SD=9.25) | Middle-aged male suicides from 2006–2009, whose bereaved consulted the prefectural MH Welfare Centres (informant report). | Interviews with the closest bereaved | Help-seeking from health professionals (including consultation or treatment from physicians, other medical professionals or psychiatrists) within 12 months before death. | Financial/job problems (unmanageable debt) |  |
| Kavalidou, McPhedran ) (2015) | Queensland, Australia | Total: 1375  Farming/agriculture: 212  Other occupations: 1163 | Farming or agriculture: M=43.0, SD=15.1  Other occupation: M=37.5, SD=12.6 | Male suicides who resided in rural areas (QSR). Time period not specified. | QSR | Contact with a MH professional within 3 months before death; current or previous treatment for a psychiatric condition. | Occupation |  |
| King (2001) | Wessex, United Kingdom | 1457 | 15+ | Suicides from 1988-1993 identified by ICD-9 codes E950–959 (Wessex Coroners' records; Office of Population Censuses and Surveys). Included death by injury of undetermined intent (ICD-9 codes and E980–989, excluding E9888). | Coronial records | Contact with a specialist psychiatric service in the 12 months before death. | Sex; contact with medical practitioners (on day of, and week leading up to death); previous suicide attempt; method of suicide |  |
| King, Schlichthorst (2020) | Victoria, Australia | 461 | 65+ | Older suicides from 2009–2015 (Victorian Suicide Register of the Coroner’s Court of Victoria). Included cases where the circumstances of death were consistent with suicide. | Coronial records | Contact with a GP for MH reasons within 6 weeks and 12 months before death; contact with another professional for MH reasons within 6 weeks and 12 months before death. | Sex; age |  |
| Law, Wong (2010) | Hong Kong, China | 119 | 15–59 (M=39.8, SD=11) | Suicides with at least one psychiatric diagnosis between September 2003 and December 2005; identified by a coronial verdict of suicide and ICD-10 codes X60–X84 (Hong Kong Coroner’s records). Cases were those for whom psychological autopsy data were available. | Interviews with next-of-kin of suicide cases | Contact with a psychiatrist within 6 months before death. | Sex; age; employment status; marital status; income; education level; living situation; schizophrenia and related disorders; past suicidal behaviour; perceived suicide risk; method of suicide; impulsivity; social problem-solving skills; social support; financial/job problems; contact with GP |  |
| Law, Wong (2015) | Hong Kong, China | Total: 115  Diagnosis: 86  No diagnosis: 29 | Diagnosis: M=34.1, SD=11.64  No diagnosis: M= 39.9, SD= 11.06 | Suicides without a psychotic diagnosis between September 2003 and December 2005, identified by a coronial verdict of suicide and ICD-10 codes X60–X84 (Hong Kong Coroner’s records). Cases were those for whom psychological autopsy data were available. | Interviews with next-of-kin of suicide cases | Treatment for MH problems in clinical psychology or social services; consultation with a psychiatrist within 6 months before death. | Any MH diagnosis |  |
| Lee, Lin (2008) | Taiwan | 19426 | 15+ | Suicides from 1998–2004 identified by ICD-9 codes E950– E959 (Taiwanese Department of Health). | Predictors: Taiwanese Department of Health  MHS use: NHID | MHS use (including outpatient and inpatient MHS provided by psychiatrists, psychologists, social workers, occupational therapists and nurses) within 12 and 1 month/s before death. | Sex; age |  |
| Loh, Tai (2012) | Singapore | 188 | 10-24 | Youth Singaporean resident suicides from 2000–2004 (Singapore Coroner’s Court) | Coronial records | Lifetime contact with mental health professionals | Sex; age; ethnicity; method of suicide; employment status; previous suicide attempts; family history of suicide; suicide note; identified stressors |  |
| Malchy, Enns (1997) | Manitoba, Canada | Total: 1029  Aboriginal: 227  Non-aboriginal: 802 | All ages (range and mean not provided) | All suicides from 1988–1994 (Office of the Chief Medical Examiner of Manitoba). | Office of the Chief Medical Examiner of Manitoba | Previous psychiatric service receipt. | Sex; age; ethnicity (i.e., indigenous vs. non-indigenous) |  |
| McLone, Kouvelis (2016) | Illinois, United States | Total: 386  Aged 15-19: 153  Aged 20-24: 233 | 15-24 | Youth suicides from 2005–2010 (IVDRS). | IVDRS | MH/substance abuse treatment, including pharmacotherapy and counselling, within 2 months before death. | Age |  |
| McPhedran and De Leo (2013) | Queensland, Australia | Total: 3203  Rural: 1418  Urban: 1785 | 18+  Rural: M=44.5, SD=17.9  Urban: M=42.8, SD=17.4 | Adult male suicides from 1990–2008 (QSR). | QSR | Contact with a MH professional for a psychiatric condition within 3 months before death. | Location |  |
| Niederkrotenthaler, Logan (2014) | United States | Total: 57877  MH treatment: 16471  No MH treatment: 41406 | 18+ | Adult suicides from 2005–2010 (NVDRS). | NVDRS | MH/substance abuse treatment, including pharmacotherapy and counselling, within 2 months before death. | Sex; age; ethnicity; marital status; living situation; depression; alcohol/substance use; past suicidal behaviour; method of suicide; physical health problem; financial/job problems; relationship/family problems; legal problems; perpetrator/victim of violence; recent loss |  |
| Pennington, Ylitalo (2021) | United States | Total: 193152  Firefighters: 722  Non-firefighters: 192430 | M=47.25, SD=17.48 | All suicides from 2003-2017 (NVDRS). | NVDRS | MH/substance abuse treatment, including pharmacotherapy and counselling, within 2 months before death. | Occupation; method of suicide |  |
| Persons, Hefti (2019) | Iowa, United States | 657 | 10–90 (M=43, SD=17) | Suicides autopsied by the University of Iowa Hospitals and Clinics between July 2003 and June 2018. | University of Iowa autopsy reports | Prior contact with MHS, either through inpatient, outpatient or emergency room visits. | Sex; age |  |
| Pitkälä, Isometsä (2000) | Finland | Total: 1397  Age 65+: 211  Age <65: 1186 | 15+ | All suicides between April 1987 and March 1988 (Finnish National Suicide Prevention Project). | Finnish National Suicide Prevention Project (includes information from medical records and interviews with family members and attending health care personnel) | Receipt of psychiatric services (including inpatient and outpatient services, and private psychiatrists) within 12 months before death. | Sex; age; method of suicide |  |
| Renaud, Berlim (2009) | Quebec, Canada | 55 | 11–18 (M=16.8, SD=1.5) | Youth suicides whose families were recruited between January 2000 and May 2003 (Province of Quebec coronial records). Time period of death not specified. | Interviews with a key respondent best acquainted with the decedent. | Contact with MHS and contact with psychiatric services within 1 and 12 month/s before death. | Sex** |  |
| Renaud, Berlim (2010) | Quebec, Canada | Total: 55  Same sex orientation: 4  Without same sex orientation: 51 | 11–18 (M=16.8, SD=1.5) | Youth suicides whose families were recruited between January 2000 and May 2003 (Province of Quebec coronial records). Time period of death not specified. | Interviews with a key respondent best acquainted with the decedent. | Contact with a psychiatrist within 1 and 12 month/s before death. | Sexual orientation |  |
| Ribeiro, Gutierrez (2017) | United States | 569 | Not specified | Soldiers who died by suicide and were on active duty any time from 2004-2009 (Armed Forces Medical Examiner’s office). | Historical Administrative Data Study of Army Study to Assess Risk and Resilience in Servicemembers | MHS visits (including inpatient visits with a documented ICD mental or behavioural disorder, outpatient visits with a MH specialist and MH encounters with a general medical provider) within 4 weeks and 12 months before death. | Sex; age; ethnicity; marital status |  |
| Roberts (2019) | United States | Total: 870  Law enforcement: 299  Firefighters: 151  Army: 420 | Law enforcement: M=40.4, SD=9.3  Firefighters: M=38.8, SD=11.6  Army: M=30.7, SD=10.8" | Law enforcement officers, firefighters and army members who died by suicide from 2003–2012 (NVDRS). | NVDRS | MH/substance abuse treatment, including pharmacotherapy and counselling, within 2 months before death. | Occupation |  |
| Rodway, Tham (2020) | United Kingdom | 595 | 10-19 | Youth suicides identified by ICD-10 codes X60–X84 from 2014–2016 (English and Welsh Office of National Statistics, National Records of Scotland, Northern Ireland Statistics and Research Agency). Included probable suicides (death by injury of undetermined intent ICD-10 codes Y10–Y34, excluding Y33.9, Y87.0, and Y87.2). | Predictors: reported by family or friends during an official investigation  MHS use: National Confidential Inquiry into Suicide in MH data | MHS contact within 12 months before death. | Sex |  |
| Runeson (1992) | Gothenburg, Sweden | 58 | 15-29.  No MHS use: M=22.6  Outpatients: M=21.8  Inpatients: M=24.5 | Youth suicides from 1984-1987 identified by ICD codes E950-959 (Gothenburg Department of Forensic Medicine and Public Health Committee). Included deaths by injury of undetermined intent (ICD codes E980-989) screened as probable suicide. | Predictors: interviews with close relatives, partners, landlords or close friends; retrospective psychiatric assessments by 2 psychiatrists.  MHS use: local medical registers | Contact with outpatient or inpatient MHS over lifetime. | Sex; academic performance; living situation (i.e., whether decedent had left home); employment status; family history of substance abuse; family history of parasuicide; family history of psychiatric contact; time from initial onset of suicidal behaviour; past suicidal behaviour; disclosure of suicide ideation to others; method of suicide; suicide note; schizophrenia; major depression; adjustment disorder; borderline personality disorder; substance abuse disorder |  |
| Ryan, Ghahramanlou-Holloway (2020) | United States | 800 | Not specified | Military personnel who died by suicide from 2002–2008 (randomly selected from US military’s Medical Mortality Registry). | Predictors: US military’s Medical Mortality Registry  MHS use: Armed Forces Health Surveillance Center data | MH visits (including any health service visit that included an ICD-9 MH code or substance abuse code) over lifetime and within 7 days before death. | Sex; age; ethnicity; occupation (within military) |  |
| Salib and Green (2003) | Cheshire, United Kingdom | 200 | 60-86 (M=71, SD=8) | Older suicides from 1989–2001 (Coroner’s office of Cheshire). | Coroner's office of Cheshire | Whether subject was known to psychiatric services. | Sex; marital status; living situation; parental status; any MH diagnosis; past suicidal behaviour; method of suicide; contact with GP |  |
| Schaffer, Sinyor (2016) | Toronto, Canada | 2835 | All ages (range and mean not provided) | All suicides from 1998–2011 (OCCO). | OCCO | MHS contact (including a MH-related primary care outpatient physician contact, an outpatient psychiatric contact, a MH-related ED visit or a MH-related hospitalisation) within 12 months before death. | Sex; age; marital status; living situation; schizophrenia and related disorders; bipolar disorder; past suicidal behaviour; method of suicide; presence of a suicide note; location of death; any stressor; physical health problems; financial/job problems; relationship/family problems; legal problems; recent loss; immigration problems |  |
| Schaffer, Sinyor (2014) | Toronto, Canada | 2886 | All ages (range and mean not provided) | All suicides from 1998–2011 (OCCO). | OCCO | Contact with psychiatry or ER services within 1 week before death. | Bipolar disorder |  |
| Schmutte and Wilkinson (2020) | United States | 26884 | 65+ | Older suicide from 2003–2016 (NVDRS). | NVDRS | Past MH or substance abuse treatment. | Any MH diagnosis |  |
| Searles, Valley (2014) | United States | 17504 | 6-101 | All suicides with known circumstances surrounding suicide from 2006–2008 (NVDRS). | NVDRS | MH/substance abuse treatment, including pharmacotherapy and counselling, within 2 months before death, and over lifetime. | Location |  |
| Séguin, Boyer (2010) | Montreal, Canada | Total: 122  Problem gamblers: 49  Not problem gamblers: 73 | Problem gamblers: M=44.7  Not problem gamblers: M=43.5 | Suicides for whom psychological autopsy data were available (Quebec Coroner's Office and Montreal Central Morgue). Time period not specified. | Predictors and MHS use: interviews with next-of-kin  Predictors: retrospective psychiatric assessments by 2 psychiatrists.  MHS use: inpatient and outpatient hospital records; expert panel review. | Contact with specialised MHS (psychiatrists, psychologists, nurses, crisis centre workers, addiction treatment and emergency departments) within lifetime and 1 and 12 month/s before death. | Problem gambling |  |
| Shahpesandy, Oakes (2014) | Isle of Wight, United Kingdom | Total: 68  Service users: 53  Non-service users: 15 | 17-87  Service users: M=44.4  Non-service users: M=45.9 | Resident suicides identified by ICD-10 codes X60–X84 between January 2006 and December 2009 (Isle of Wight coronial records). Included open verdicts screened as probable suicide. | Medical case records; coroner's reports; suicide notes | Receipt of MHS within 2 years before death. | Employment status; marital status; education level; depression; anxiety; schizophrenia and related disorder; bipolar disorder; personality disorder; alcohol/substance use; past suicidal behaviour; method of suicide; family history of mental illness; any recent stressor; physical health problem; financial/job problems; relationship/family problems; legal problems; recent loss; housing problems |  |
| Stark, Huc (2012) | Scottish Highlands, United Kingdom | 177 | 15+ | Resident suicides from 2001–2004 (General Registrar Office for Scotland). Included deaths by injury of undetermined intent. | Predictors: General Registrar Office for Scotland  MHS: health service databases (including general hospital, psychiatric and GP case notes) | MHS use over lifetime, and within 1 and 12 month/s before death. | Location; contact with any health service |  |
| Sundqvist‐Stensman (1987) | Uppsala, Sweden | 523 | All ages (range and mean not provided) | All suicides from 1977–1984 (Department of Forensic Medicine in Uppsala autopsy registers). Included death by injury of undetermined intent. | Predictors: Department of Forensic Medicine in Uppsala autopsy registers; police reports; GP records; Social Welfare Bureau records  MHS use: psychiatric hospital records | Treatment as in- or outpatients at either of the two psychiatric hospitals in the county over lifetime. | Marital status; location; depression; alcohol/substance abuse; past suicidal behaviour; disclosure of suicide intent; method of suicide; presence of suicide note; physical health problem; financial/job problems; relationship/family problems; recent loss |  |
| Suso-Ribera, Mora-Marín (2018) | Castellon, Spain | 312 | 15+ | All suicides from 2009–2015 (Institute of Forensic Medicine of Castellon records) | Institute of Forensic Medicine of Castellon records | Specialised MHS use within 1 year before death; GP visit for psychiatric reasons within 1 year before death; psychiatric hospitalisation over lifetime. | Location |  |
| Sveticic, Milner (2012) | Queensland, Australia | Total: 7126  Aboriginal and Torres Strait Islander: 471  Non-indigenous: 6655 | All ages (range and mean not provided) | All suicides with known ethnicity from 1994–2007 (QSR) | QSR | Contact with MHS over lifetime and within 3 months before death. | Sex; ethnicity (indigenous vs. non-indigenous); marital status; location; alcohol/substance use; past suicidal behaviour; contact with health services for physical illness |  |
| Sweeney, Fontanella (2020) | Ohio, United States | 1273 | 65+ | Older suicides from 2012–2015 (OH-VDRS). | OH-VDRS | Contact with behavioural health services (including seeing a psychiatrist, psychologist, medical doctor, therapist or other counsellor for MH or substance abuse problem; receiving a prescription for a psychiatric medicine; attending anger management classes; residing in an inpatient or halfway house for MH or substance abuse problems; or involvement with alcohol or narcotics anonymous) within 2 months before death. | Sex; age; ethnicity; marital status; location; occupation (i.e., military service); depression; alcohol/substance use; past suicidal behaviour; method of suicide; physical health problem; financial/job problems; family/ relationship problems; perpetrating violence; recent loss |  |
| Vassilas and Morgan (1997) | Avon, England | 144 | 17-85 (M=43, SD=18) | All suicides from March 1990–November 1991 (Her Majesty's Coroner's data for the County of Avon). Included open verdicts and verdicts of death by accident/misadventure screened as probable suicide. | Predictors: Her Majesty's Coroner's data for the County of Avon  MHS use: GP notes; interviews with GPs; psychiatric and general medical hospital records; data from the Avon Social Services and Avon Council on Alcoholism and Drugs | Lifetime contact with a psychiatrist; contact with MH services within 4 weeks before death. | Sex; age |  |
| Waitz‐Kudla, Daruwala (2019) | United States | N = 147 for political analyses  N = 218 for religion analyses | M=36.19, SD=16.05 | People who died by suicide whose bereaved were recruited from Facebook, support groups, and suicide-related listservs. Time period of death not specified. | Interviews with bereaved | Use of medication and/or contact with a health provider for any mental illness during a 'previous period' in their life, and in the 'weeks and days' preceding death. If help-seeking endorsed for one or both variables, decedents were categorised as having sought help. | Religion; political views | |
| Windfuhr, While (2008) | United Kingdom | 1722 | 10-19 | Youth suicides identified by ICD-10 codes X60-X84 and X87 (excluding X87.2) from 1997–2003 (English and Welsh Offices for National Statistics, Northern Irish and Scottish General Register's Offices). Included open verdicts identified by ICD-10 codes Y10-Y34 (excluding Y33.9). | Offices for National Statistics; General Register’s Offices; National Confidential Inquiry into Suicide and Homicide by People with Mental Illness | Contact with MHS (including outpatient and inpatient services) within 12 months before death. | Sex |  |
| Wong, Wang (2017) | United States | Total: 92658  Asian and Pacific Islander Americans: 1294  White Americans: 91364 | All ages (range and mean not provided) | White, Asian and Pacific Islander suicides from 2003–2013 (NVDRS). | NVDRS | MH/substance abuse treatment, including pharmacotherapy and counselling, within 2 months before death. | Sex; age; ethnicity |  |
| Zaheer, Jacob (2018) | Ontario, Canada | Total: 5650  Schizophrenia: 663  No schizophrenia: 4987 | All ages (range and mean not provided) | All suicides from 2008–2012 (Office of the Registrar–Deaths Database ICD codes, NACRS and OMHRS databases). | ICES; Ontario Health Insurance Plan database; NACRS; OMHRS; Canadian Institutes of Health Information Discharge Abstract Database | MHS use (including outpatient psychiatrist, MH-related primary care, and MH-related ED visits and hospitalisations) within 30 days before death. | Schizophrenia and related disorders |  |

*Notes*: COVDRS = Colorado Violent Death Reporting System, ED = emergency department, GP = general practitioner, ICD = Classification of Diseases, ICES = Institute for Clinical and Evaluative Sciences, IVDRS = Illinois Violent Death Reporting System, MH = mental health, MHS = mental health services, NACRS = National Ambulatory Care Reporting System, NHID = National Health Insurance Database, NVDRS = National Violent Death Reporting System, OCCO = Office of the Chief Coroner for Ontario, OH-VDRS = Ohio Violent Death Reporting System, OMHRS = Ontario Mental Health Reporting System, QSR = Queensland Suicide Register. *Cases where the same data sources were used or data sources were not clearly differentiated for the predictors and MHS use, are reported together. **Renaud et al. (2009) also examined the relationship between MH diagnosis and non-receipt of MHS. However, as their clustering of diagnoses was not comparable to other studies, this data was excluded from the current review.

**Supplementary Table 4. Quality assessment ratings of all included studies.**

|  | Item 1.  Measurement of condition | | Item 2.  Exposure variables | Item 3. Outcome measure | Item 4.  Confounding factors |
| --- | --- | --- | --- | --- | --- |
| Ahmedani, Simon (2014) | + | + | | + | - |
| Ali, Rockett (2021) | / | + | | / | + |
| Arnautovska, McPhedran (2015) | + | + | | / | + |
| Bakst, Braun (2014) | + | + | | - | / |
| Betz, Krzyzaniak (2011) | + | + | | / | + |
| Booth, Briscoe (2000) | / | + | | / | - |
| Chang, Liao (2009) | + | + | | + | - |
| Chang, Lai (2012) | + | + | | + | + |
| Chen, Liao (2009) | + | + | | + | ? |
| Cheung, Douwes (2017) | + | + | | / | - |
| Cho, Kang (2013) | + | + | | + | - |
| Choi, DiNitto (2017) | + | + | | / | + |
| Choi, DiNitto, Marti, Conwell (2019) | + | + | | / | + |
| Choi, DiNitto, Marti (2019) | + | + | | / | - |
| Coope, Donovan (2015) | + | + | | / | - |
| De Leo, Draper (2013) | ? | - | | - | + |
| Foster, Gillespie (1997) | + | + | | / | - |
| Frei, Bucher (2013) | / | + | | + | + |
| Giupponi, Pycha (2014) | + | / | | - | + |
| Grigoriadis, Wilton (2017) | + | + | | + | - |
| Haaland, Bjørkhold (2017) | + | + | | + | - |
| Hamdi, Price (2008) | / | + | | / | + |
| Hayward, Zubrick (1992) | + | + | | / | - |
| Hintikka, Lehtonen (1997) | + | + | | / | - |
| Ho, Ho (2014) | + | + | | / | - |
| Isometsa, Henriksson (1994) | + | - | | / | - |
| Joe, Marcus (2007) | + | + | | - | + |
| Jung, Lee (2019) | ? | + | | - | - |
| Kameyama, Matsumoto (2011) | - | - | | - | - |
| Kavalidou, McPhedran ) (2015) | + | + | | / | - |
| King (2001) | / | / | | / | - |
| King, Schlichthorst (2020) | + | + | | / | - |
| Law, Wong (2010) | + | / | | - | + |
| Law, Wong (2015) | + | - | | - | - |
| Lee, Lin (2008) | + | + | | + | + |
| Loh, Tai (2012) | + | / | | / | + |
| Malchy, Enns (1997) | + | + | | / | + |
| McLone, Kouvelis (2016) | + | + | | / | - |
| McPhedran and De Leo (2013) | + | + | | / | / |
| Niederkrotenthaler, Logan (2014) | + | + | | / | + |
| Pennington, Ylitalo (2021) | + | + | | / | - |
| Persons, Hefti (2019) | + | + | | / | - |
| Pitkälä, Isometsä (2000) | + | + | | / | - |
| Renaud, Berlim (2009) | + | / | | - | + |
| Renaud, Berlim (2010) | + | - | | - | + |
| Ribeiro, Gutierrez (2017) | + | + | | + | - |
| Roberts (2019) | + | + | | / | + |
| Rodway, Tham (2020) | / | + | | + | + |
| Runeson (1992) | + | / | | + | - |
| Ryan, Ghahramanlou-Holloway (2020) | + | + | | + | ? |
| Salib and Green (2003) | + | + | | / | + |
| Schaffer, Sinyor (2016) | + | + | | + | + |
| Schaffer, Sinyor (2014) | + | + | | / | + |
| Schmutte and Wilkinson (2020) | + | + | | / | + |
| Searles, Valley (2014) | + | + | | / | + |
| Séguin, Boyer (2010) | + | - | | / | + |
| Shahpesandy, Oakes (2014) | + | + | | / | - |
| Stark, Huc (2012) | / | + | | + | - |
| Sundqvist‐Stensman (1987) | / | + | | + | - |
| Suso-Ribera, Mora-Marín (2018) | + | + | | + | + |
| Sveticic, Milner (2012) | + | / | | / | + |
| Sweeney, Fontanella (2020) | + | + | | / | + |
| Vassilas and Morgan (1997) | + | + | | + | - |
| Waitz‐Kudla, Daruwala (2019) | - | - | | - | + |
| Windfuhr, While (2008) | / | + | | + | - |
| Wong, Wang (2017) | + | + | | / | + |
| Zaheer, Jacob (2018) | + | + | | + | / |
|  |  |  | |  |  |
| % Adequate | 80.60 | 79.10 | | 29.85 | 46.27 |
| % Partial | 13.43 | 10.45 | | 53.73 | 4.48 |
| % Poor | 2.99 | 10.45 | | 16.42 | 46.27 |
| % Unclear | 2.99 | 0.00 | | 0.00 | 2.99 |

*Note.* + adequate; / partial; - poor; ? unclear.

**Supplementary materials: References included in the systematic review, but not the main text**

Grigoriadis S, Wilton AS, Kurdyak PA, Rhodes AE, VonderPorten EH, Levitt A, et al. Perinatal suicide in Ontario, Canada: A 15-year population-based study. Canadian Medical Association Journal. 2017;189(34):E1085-e92.

Renaud J, Berlim MT, Begolli M, McGirr A, Turecki G. Sexual orientation and gender identity in youth suicide victims: An exploratory study. The Canadian Journal of Psychiatry. 2010;55(1):29-34.

Séguin M, Boyer R, Lesage A, McGirr A, Suissa A, Tousignant M, et al. Suicide and gambling: Psychopathology and treatment-seeking. Psychology of Addictive Behaviors. 2010;24(3):541-7.
